# Trends in Kidney Stone Prevalence Among United States Adults With Diabetes: A Cross‐Sectional Study From the NHANES Database, 2007–2020

**DOI:** 10.1155/jdr/4305574

**Published:** 2026-04-22

**Authors:** Ziqiao Wang, Junlong Huang, Ruixiang Luo, Fanyu Wu, Ziliang Deng, Zheng Liu, Wenshuang Li, Xiangfu Zhou, Bolong Liu

**Affiliations:** ^1^ Department of Urology, The Third Affiliated Hospital of Sun Yat-sen University, Guangzhou, Guangdong, China, zssy.com.cn; ^2^ Department of Urology, People’s Hospital of Yingde City, Yingde, Guangdong, China

**Keywords:** diabetes, kidney stone, NHANES, prevalence, trend

## Abstract

**Background:**

This study aimed to estimate the trends in the prevalence of kidney stones among adults with diabetes in the United States from 2007 to 2020 and to examine sex‐ and race/ethnicity‐specific differences.

**Methods:**

This study analyzed 31,116 adult participants from the National Health and Nutrition Examination Survey (NHANES) 2007–2020. Survey‐weighted multivariable logistic regression was used to examine the association between diabetes and kidney stones, and survey‐weighted linear regression was used to evaluate temporal trends overall and across subgroups defined by sex and race/ethnicity.

**Results:**

Diabetes was associated with higher odds of kidney stones (OR = 1.63, *p* < 0.001). Between 2007 and 2020, the prevalence of both diabetes and kidney stones increased significantly among US adults (both *p* for trend <0.05). In subgroup analyses by sex and race, diabetes prevalence increased significantly over time among men and non‐Hispanic Whites (both *P* for trend <0.05). Among adults with diabetes, kidney stone prevalence increased from 14.3% to 16.1% (*p* for trend = 0.446). Among men with diabetes, kidney stone prevalence changed from 16.6% to 20.2% (*p* for trend = 0.527), whereas among women with diabetes, it changed from 11.8% to 12.8% (*p* for trend = 0.848). By race/ethnicity, among diabetic Hispanics, kidney stone prevalence significantly increased from 8.7% to 14.8% (*p* for trend = 0.017). Non‐Hispanic White adults with diabetes had the highest kidney stone prevalence (17.4% to 21.7%, *p* for trend = 0.468), and non‐Hispanic Black adults with diabetes changed from 7.7% to 6.9% (*p* for trend = 0.775).

**Conclusion:**

From 2007 to 2020, the diabetes prevalence significantly increased. Kidney stone prevalence increased significantly among Hispanic adults with diabetes and remained high among men with diabetes and non‐Hispanic White adults with diabetes. These subgroups may warrant targeted prevention and clinical attention.

## 1. Introduction

Kidney stones are a common urological condition in the United States, affecting ~10% of adults, and the prevalence continues to rise [1]. This condition imposes a substantial health burden and is associated with reduced quality of life [2, 3]. Among various risk factors, diabetes has been associated with kidney stones, potentially through metabolic abnormalities such as dysregulation of calcium and uric acid [4–6]. Diabetes is also highly prevalent worldwide. In 2021, the International Diabetes Federation estimated that one in 10 adults aged 20–79 years worldwide had diabetes, and that ~33.2 million people in the United States were living with diabetes [7]. Recent studies indicate that both diabetes and kidney stone prevalence have increased in the general population [7, 8]. Although the association between diabetes and kidney stones is well documented [9], temporal trends in kidney stone prevalence among adults with diabetes remain incompletely characterized.

To address this gap, we used data from the National Health and Nutrition Examination Survey (NHANES), conducted by the National Center for Health Statistics within the Centers for Disease Control and Prevention. NHANES employs a multistage, probability sampling design to assess the health and nutritional status of the noninstitutionalized US civilian population [10]. Using this nationally representative dataset, we analyzed temporal trends in the prevalence of kidney stones among US adults with diabetes from 2007 to 2020, explored factors associated with kidney stones, and evaluated the related public health implications. Given the growing populations affected by both diabetes and kidney stones, clarifying these trends is vital for developing effective prevention and management strategies to address this increasing public health concern.

## 2. Materials and Methods

### 2.1. Data Sources

This study utilized data from the NHANES to assess trends in the prevalence of kidney stones among individuals with diabetes. We conducted a cross‐sectional analysis using data from six NHANES cycles from 2007 to 2020. The combined dataset for this period initially included 66,148 participants. Participants were excluded based on the following criteria: age under 20 years, missing data for diabetes status or kidney stones, or incomplete covariate information. The final sample comprised 31,116 participants, including 5364 individuals with diabetes and 25,752 individuals without diabetes. A flowchart detailing participant enrollment is provided in Supporting Information 1: Figure S1. This study followed the Strengthening the Reporting of Observational Studies in Epidemiology (STROBE) reporting guideline for cross‐sectional studies.

### 2.2. Assessment of Diabetes, Kidney Stones, and Covariates

Diabetes was defined as a self‐reported diagnosis, fasting blood glucose (FBG) ≥126 mg/dL, or HbA1c ≥6.5% [11]. NHANES does not include a validated classification to reliably distinguish type 1 from type 2 diabetes. Accordingly, we analyzed diabetes as an overall adult diabetes status. Kidney stones were identified by a positive response to the question “Ever had kidney stones?” Participants who answered “yes” were considered to have a history of kidney stones.

Age was grouped into three categories: 20–39, 40–59, and ≥60 years. Sex was classified as men or women. Race was categorized into four groups: Hispanic, non‐Hispanic White, non‐Hispanic Black, and other. Body mass index (BMI) was categorized as <25, 25–30, or ≥30 kg/m^2^. Abdominal obesity was defined as a waist circumference ≥102 cm for men or ≥88 cm for women [12]. Smoking status was determined by whether participants had smoked at least 100 cigarettes in their life. Alcohol use was defined as having consumed at least 12 drinks of any alcoholic beverage in their lifetime. In an expanded‐adjustment sensitivity analysis, we additionally considered dietary intake, kidney function, serum uric acid, socioeconomic status, and glucose‐lowering medication use. Dietary intake variables (total energy, total water, sodium, calcium, protein, and total sugars) were obtained from NHANES 24 h dietary recall data and summarized as the mean of two recall days when available (otherwise using the single available day). Kidney function was assessed by estimated glomerular filtration rate (eGFR), calculated from serum creatinine using the CKD‐EPI 2021 equation. Serum uric acid was obtained from laboratory measurements. Socioeconomic status was represented by the poverty income ratio (PIR). Glucose‐lowering medication use was defined as current use of insulin and/or oral hypoglycemic agents (yes/no).

### 2.3. Statistical Analysis

This study used NHANES sampling weights and design variables to estimate the prevalence of diabetes and kidney stones, ensuring nationally representative estimates for the US noninstitutionalized population.

We used weighted multivariable logistic regression to assess the association between diabetes and kidney stones. A directed acyclic graph (DAG) is provided in Supporting Information 1: Figure S2 to illustrate the rationale for covariate selection in this analysis. We constructed sequential models: Model 1 (unadjusted); Model 2 (primary adjusted model) adjusted for age group, sex, race/ethnicity, BMI category, alcohol use, abdominal obesity, and smoking status; and Model 3 (expanded adjustment; sensitivity analysis) further adjusted for dietary intake variables (total energy, total water, sodium, calcium, protein, and total sugars), eGFR, serum uric acid, PIR, and glucose‐lowering medication use (insulin and/or oral hypoglycemic agents; yes/no). Model 3 was conducted as an expanded‐adjustment sensitivity analysis in the subset with available data for the additional covariates.

Prevalence estimates across NHANES cycles were age‐standardized to the 2020 US Census population using the age groups 20–39, 40–59, and ≥60 years. Temporal trends were evaluated by treating NHANES cycles as a continuous variable in survey‐weighted regression models, with trend significance assessed by *p* for trend. Primary analyses were prespecified to evaluate the overall diabetes–kidney stone association (adjusted for key covariates) and the overall time trend in kidney stone prevalence among adults with or without diabetes. Subgroup analyses by sex and race/ethnicity were conducted to explore potential heterogeneity and were considered secondary and exploratory. No adjustments were made for multiple comparisons. Therefore, findings from secondary analyses should be interpreted cautiously as exploratory.

All analyses were conducted using R software. Figures were generated using GraphPad Prism. All tests were two‐sided, and *p* < 0.05 was considered statistically significant.

## 3. Results

### 3.1. Baseline Characteristics of Participants

Table 1 presents the baseline characteristics of participants with or without diabetes in the NHANES 2007–2020 combined cycles. Among participants with diabetes, 8.8% were aged 20–39 years, 38.8% were aged 40–59 years, and 52.4% were aged ≥60 years. Among participants with diabetes, 52.4% were men and 47.6% were women. By race/ethnicity among participants with diabetes, 60.5% were non‐Hispanic White, 16.0% were Hispanic, and 14.6% were non‐Hispanic Black. Kidney stone prevalence was higher among participants with diabetes than among those without diabetes (17.4% and 8.7%, respectively).

**Table 1:** Baseline characteristics of the weighted study population, NHANES 2007–2020.

Characteristics	Overall	No diabetes	Diabetes		*p*
	Weighted *N* = 198,504,253	Weighted *N* = 173,177,976	Weighted *N* = 25,326,277		
Age, years, *n* (%)					
<40	73,060,009 (36.8)	70,828,348 (40.9)	2,231,661 (8.8)		<0.001
40–59	72,964,275 (36.8)	63,130,247 (36.5)	9,834,027 (38.8)		
≥60	52,479,969 (26.4)	39,219,381 (22.6)	13,260,588 (52.4)		
Sex, *n* (%)					
Men	97,104,583 (48.9)	83,839,624 (48.4)	13,264,958 (52.4)		<0.001
Women	101,399,670 (51.1)	89,338,352 (51.6)	12,061,318 (47.6)		
Race, *n* (%)					
Hispanic	28,714,477 (14.5)	24,666,801 (14.2)	4,047,676 (16.0)		<0.001
Non‐Hispanic White	132,735,455 (66.9)	117,405,229 (67.8)	15,330,226 (60.5)		
Non‐Hispanic Black	21,711,147 (10.9)	18,011,830 (10.4)	3,699,317 (14.6)		
Other race	15,343,174 (7.7)	13,094,117 (7.6)	2,249,058 (8.9)		
Kidney stones, *n* (%)					
No	179,058,413 (90.2)	158,150,115 (91.3)	20,908,297 (82.6)		<0.001
Yes	19,445,840 (9.8)	15,027,861 (8.7)	4,417,979 (17.4)		

*Note:* Weighted *N* represents estimated population counts derived from NHANES survey weights. Weighted estimates were rounded to the nearest whole number; therefore, subgroup totals may not sum exactly to the overall total because of rounding.

### 3.2. Association Between Diabetes and Kidney Stones

Multivariable logistic regression showed that diabetes was associated with higher odds of kidney stone history (OR = 1.63; 95% CI, 1.41–1.88; *p* < 0.001). This association remained after adjustment for age, sex, race/ethnicity, obesity, and other covariates (Supporting Information 1: Table S1). In sensitivity analyses treating age and BMI as continuous variables, results were similar (OR = 1.58; 95% CI, 1.36–1.83; *p* < 0.001) (Supporting Information 1: Table S2). In a further sensitivity analysis with expanded adjustment for dietary intake, eGFR, serum uric acid, PIR, and glucose‐lowering medication use, diabetes remained associated with kidney stone history (OR = 1.50; 95% CI, 1.13–1.99; *p* = 0.006) (Supporting Information 1: Table S3).

### 3.3. Trends in the Prevalence of Diabetes Over Time

As shown in Figure 1A and Supporting Information 1: Table S4, the prevalence of diabetes among US adults significantly increased from 12.4% (95% CI, 11.1%–13.9%) in 2007 to 14.5% (95% CI, 13.4%–15.6%) in 2020 (*p* for trend = 0.004). Table 2 presents the annualized mean counts of characteristics among individuals with diabetes across NHANES survey cycles from 2007–2008 to 2017–2020. The annualized mean number of individuals with diabetes increased from 1,621,937 in 2007–2008 to 2,319,079 in 2017–2020.

Figure 1Trends in age‐standardized prevalence of diabetes among US adults from 2007 to 2020. (A) Trends in age‐standardized prevalence of diabetes among US adults by sex from 2007 to 2020. (B) Trends in age‐standardized prevalence of diabetes among US adults by race/ethnicity from 2007 to 2020.

**Figure:**
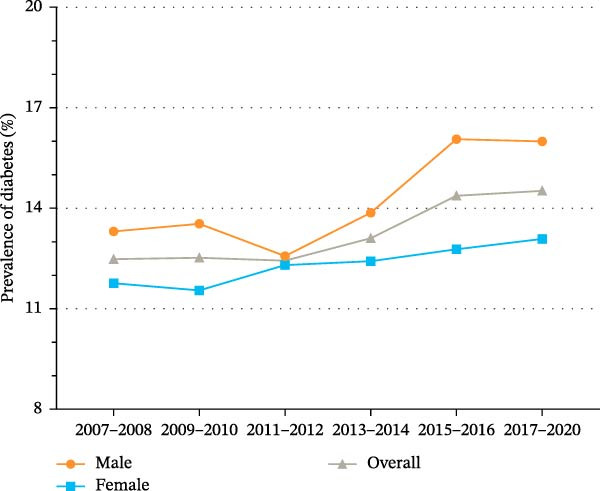
(A)

**Figure:**
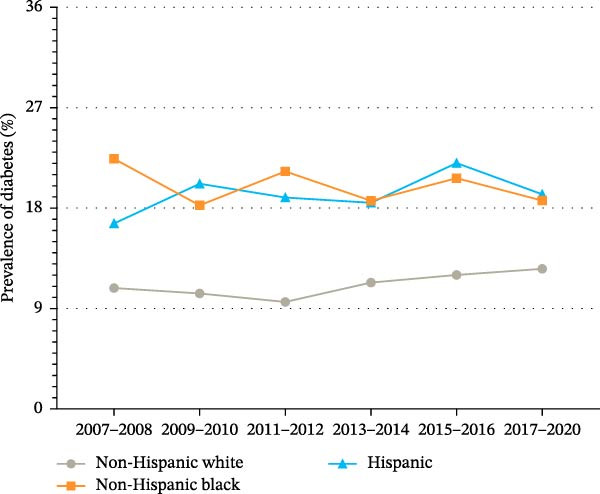
(B)

**Table 2:** Annualized mean counts and weighted percentages of characteristics among adults with diabetes across NHANES survey cycles, 2007–2020.

Characteristic	2007–2008	2009–2010	2011–2012	2013–2014	2015–2016	2017–2020	*p*
Diabetes	1,621,937	1,609,975	1,723,328	1,855,199	2,142,172	2,319,079	
Age, years, *n* (%)							
<40	177,483 (10.9)	129,009 (8.0)	142,998 (8.3)	143,465 (7.7)	222,121 (10.4)	187,972 (8.1)	0.425
40–59	672,299 (41.5)	617,727 (38.4)	724,255 (42.0)	718,929 (38.8)	821,826 (38.4)	851,237 (36.7)	
≥60	772,156 (47.6)	863,239 (53.6)	856,076 (49.7)	992,806 (53.5)	1,098,225 (51.3)	1,279,871 (55.2)	
Sex, *n* (%)							
Men	814,311 (50.2)	857,609 (53.3)	853,088 (49.5)	947,293 (51.1)	1,164,292 (54.4)	1,247,429 (53.8)	0.588
Women	807,626 (49.8)	752,366 (46.7)	870,241 (50.5)	907,906 (48.9)	977,880 (45.6)	1,071,650 (46.2)	
Race, *n* (%)							
Non‐Hispanic White	1,044,060 (64.4)	998,796 (62.0)	972,912 (56.5)	1,158,184 (62.4)	1,261,134 (58.9)	1,393,767 (60.1)	0.666
Hispanic	207,849 (12.8)	273,395 (17.0)	280,161 (16.3)	271,561 (14.6)	400,503 (18.7)	368,980 (15.9)	
Non‐Hispanic Black	297,131 (18.3)	226,092 (14.0)	289,722 (16.8)	262,183 (14.1)	302,178 (14.1)	295,221 (12.7)	
Other race	72,897 (4.5)	111,692 (6.9)	180,534 (10.5)	163,272 (8.8)	178,356 (8.3)	261,111 (11.3)	
Kidney stones, *n* (%)							
No	1,352,892 (83.4)	1,358,847 (84.4)	1,459,126 (84.7)	1,483,621 (80.0)	1,696,947 (79.2)	1,939,197 (83.6)	0.144
Yes	269,045 (16.6)	251,127 (15.6)	264,203 (15.3)	371,579 (20.0)	445,225 (20.8)	379,881 (16.4)	

*Note:* Data are presented as annualized mean counts and weighted percentages. Annualized mean counts were calculated by dividing weighted counts by 2 for the 2007–2016 survey cycles and by 3.2 for the 2017–2020 survey cycle. Counts were rounded to the nearest whole number; therefore, subgroup totals may not sum exactly to the corresponding annualized total because of rounding. *p* values were calculated using survey‐weighted tests across survey cycles. *p* < 0.05 was considered significant.

To examine the trends in the prevalence of diabetes by sex and race/ethnicity, subgroup analyses were performed (Figure 1 and Supporting Information 1: Table S4). The prevalence of diabetes among men significantly increased from 13.3% (95% CI, 11.8%–14.8%) in 2007 to 16.0% (95% CI, 14.4%–17.6%) in 2020 (*p* for trend = 0.003). Among women, the prevalence of diabetes increased from 11.8% (95% CI, 10.0%–13.6%) in 2007 to 13.1% (95% CI, 11.7%–14.5%) in 2020 (*p* for trend = 0.154). In race/ethnicity‐specific subgroup analyses (Figure 1B and Supporting Information 1: Table S4), the prevalence of diabetes among non‐Hispanic Whites increased significantly from 10.8% (95% CI, 9.0%–12.7%) in 2007 to 12.6% (95% CI, 11.1%–14.0%) in 2020 (*p* for trend = 0.035). In contrast, the prevalence of diabetes among non‐Hispanic Blacks decreased from 22.4% (95% CI, 20.0%–24.8%) in 2007 to 18.7% (95% CI, 16.9%–20.5%) in 2020 (*p* for trend = 0.126). Among Hispanics, the prevalence of diabetes increased from 16.6% (95% CI, 14.4%–18.8%) in 2007 to 19.2% (95% CI, 17.2%–21.3%) in 2020 (*p* for trend = 0.205).

### 3.4. Trends in Kidney Stone Prevalence by Diabetes Status and Sex

As shown in Figure 2A and Supporting Information 1: Table 5, the prevalence of kidney stones among US adults significantly increased from 9.3% (95% CI, 8.3%–10.3%) in 2007 to 10.1% (95% CI, 9.2%–11.1%) in 2020 (*p* for trend = 0.008). Among individuals with diabetes, the prevalence of kidney stones increased from 14.3% (95% CI, 10.9%–17.7%) to 16.1% (95% CI, 10.3%–22.0%), but the trend was not significant (*p* for trend = 0.446). Among individuals without diabetes, the prevalence of kidney stones increased significantly from 8.5% (95% CI, 7.4%–9.7%) to 9.4% (95% CI, 8.2%–10.6%) (*p* for trend = 0.028). The prevalence of kidney stones was consistently higher among individuals with diabetes than among those without diabetes across all cycles. The annualized mean number of individuals with both diabetes and kidney stones increased from 269,045 in 2007–2008 to 379,881 in 2017–2020 (Table 2).

Figure 2Trends in age‐standardized prevalence of kidney stones among US adults by diabetes status and sex from 2007 to 2020. (A) Overall trends in age‐standardized prevalence of kidney stones among US adults by diabetes status from 2007 to 2020. (B) Trends in age‐standardized prevalence of kidney stones among men by diabetes status from 2007 to 2020. (C) Trends in age‐standardized prevalence of kidney stones among the women by diabetes status from 2007 to 2020.

**Figure:**
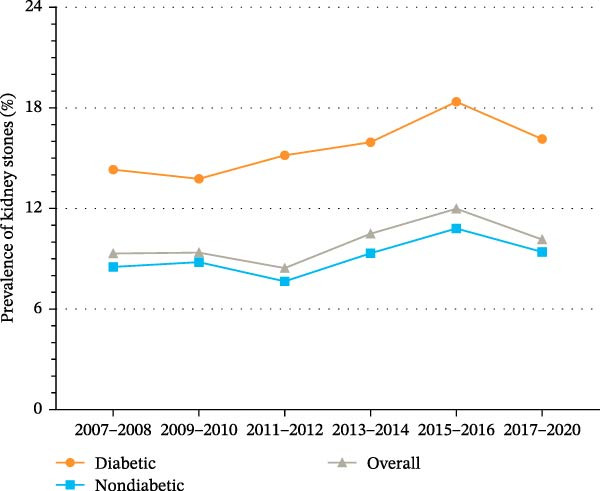
(A)

**Figure:**
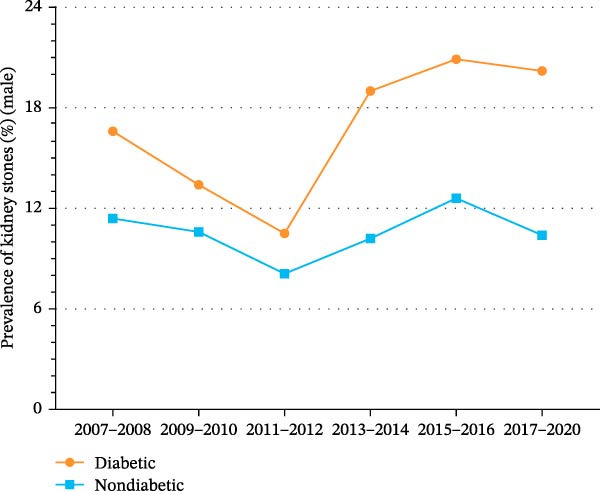
(B)

**Figure:**
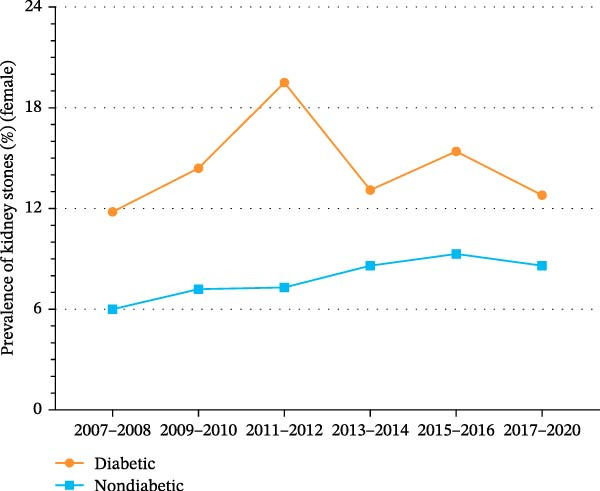
(C)

These sex‐specific trend comparisons were secondary and exploratory and are presented to describe patterns. As shown in Supporting Information 1: Figure S3A and Supporting Information 1: Table S5, the prevalence of kidney stones among men fell slightly from 12.3% (95% CI, 10.8%–13.8%) to 11.1% (95% CI, 9.5%–12.8%) (*p* for trend = 0.489). As shown in Figure 2B and Supporting Information 1: Figure S4, the prevalence of kidney stones among men with diabetes increased from 16.6% (95% CI, 12.2%–20.9%) to 20.2% (95% CI, 8.9%–31.5%) (*p* for trend = 0.527). Among men without diabetes, the prevalence of kidney stones decreased from 11.4% (95% CI, 9.7%–13.2%) to 10.4% (95% CI, 8.6%–12.2%) (*p* for trend = 0.748).

As shown in Supporting Information 1: Figure S3A and Supporting Information 1: Table S5, the prevalence of kidney stones among women significantly increased from 6.7% (95% CI, 5.4%–8.0%) to 9.2% (95% CI, 7.8%–10.6%) (*p* for trend = 0.003). As shown in Figure 2C and Supporting Information 1: Figure S4, the prevalence of kidney stones among women with diabetes increased from 11.8% (95% CI, 6.5%–17.1%) to 12.8% (95% CI, 8.2%–17.4%) (*p* for trend = 0.848). Among women without diabetes, the prevalence of kidney stones increased significantly from 6.0% (95% CI, 4.6%–7.4%) to 8.6% (95% CI, 7.1%–10.1%) (*p* for trend = 0.001).

### 3.5. Kidney Stone Prevalence Over Time by Diabetes Status and Race/Ethnicity

We evaluated kidney stone prevalence trends by race/ethnicity and diabetes status. Kidney stone prevalence rose over time across racial/ethnic groups in the overall US adult population, whereas trends among adults with diabetes differed by race/ethnicity.

As shown in Supporting Information 1: Figure S3B and Supporting Information 1: Table S5, the prevalence of kidney stones among Hispanics increased significantly from 8.4% (95% CI, 6.9%–9.8%) in 2007 to 10.1% (95% CI, 8.5%–11.7%) in 2020 (*p* for trend = 0.036). As shown in Figure 3A and Supporting Information 1: Figure S5, the prevalence of kidney stones among Hispanics with diabetes increased significantly from 8.7% (95% CI, 5.3%–12.0%) in 2007 to 14.8% (95% CI, 10.3%–19.3%) in 2020 (*p* for trend = 0.017). Among Hispanics without diabetes, the prevalence of kidney stones increased slightly from 7.9% (95% CI, 6.2%–9.6%) in 2007 to 9.1% (95% CI, 7.3%–11.0%) in 2020 (*p* for trend = 0.202).

Figure 3Trends in age‐standardized prevalence of kidney stones among US adults by diabetes status and race/ethnicity from 2007 to 2020. (A) Trends in age‐standardized prevalence of kidney stones among Hispanics by diabetes status from 2007 to 2020. (B) Trends in age‐standardized prevalence of kidney stones among non‐Hispanic Whites by diabetes status from 2007 to 2020. (C) Trends in age‐standardized prevalence of kidney stones among non‐Hispanic Blacks by diabetes status from 2007 to 2020.

**Figure:**
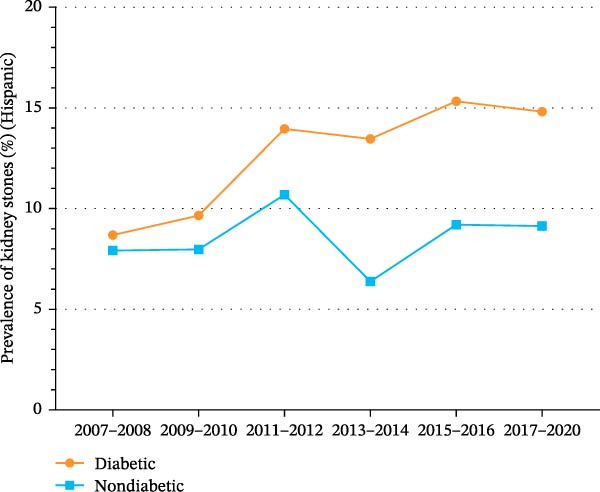
(A)

**Figure:**
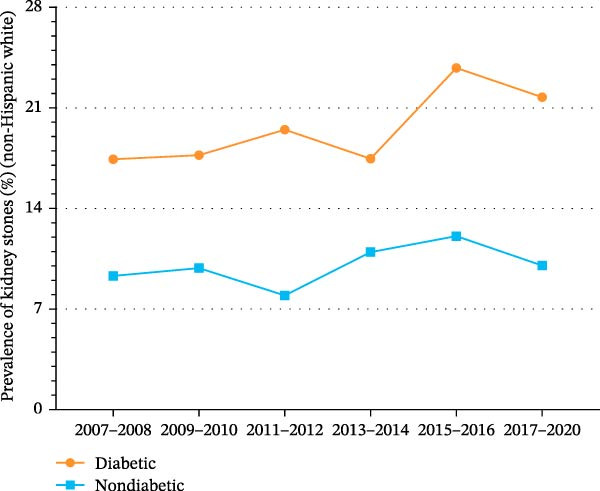
(B)

**Figure:**
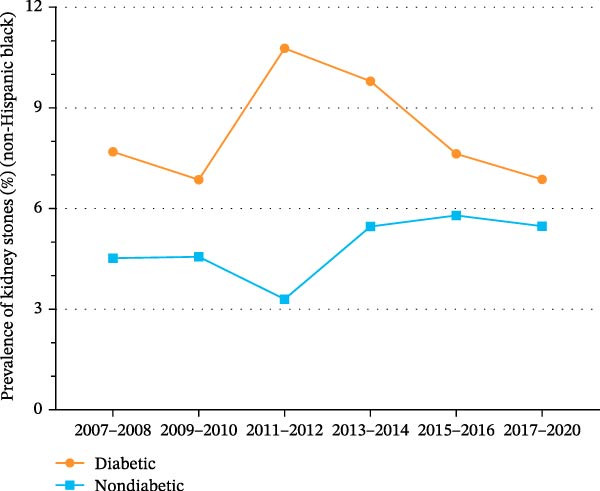
(C)

As shown in Supporting Information 1: Figure S3B and Supporting Information 1: Table S5, the prevalence of kidney stones among non‐Hispanic Whites increased significantly from 10.3% (95% CI, 9.0%–11.6%) in 2007 to 10.9% (95% CI, 9.5%–12.3%) in 2020 (*p* for trend = 0.038). As shown in Figure 3B and Supporting Information 1: Figure S5, the prevalence of kidney stones among non‐Hispanic Whites with diabetes was high and increased from 17.4% (95% CI, 12.6%–22.2%) in 2007 to 21.7% (95% CI, 10.1%–33.4%) in 2020 (*p* for trend = 0.468). Among non‐Hispanic Whites without diabetes, the prevalence of kidney stones increased slightly from 9.3% (95% CI, 7.9%–10.7%) in 2007 to 10.0% (95% CI, 8.4%–11.6%) in 2020 (*p* for trend = 0.093).

As shown in Supporting Information 1: Figure S3B and Supporting Information 1: Table S5, the prevalence of kidney stones among non‐Hispanic Blacks increased significantly from 4.8% (95% CI, 3.4%–6.3%) in 2007 to 6.1% (95% CI, 4.9%–7.3%) in 2020 (*p* for trend = 0.039). As shown in Figure 3C and Supporting Information 1: Figure S5, the prevalence of kidney stones among non‐Hispanic Blacks with diabetes decreased from 7.7% (95% CI, 3.5%–11.9%) in 2007 to 6.9% (95% CI, 3.8%–9.9%) in 2020 (*p* for trend = 0.775). Among non‐Hispanic Blacks without diabetes, the prevalence of kidney stones increased significantly from 4.5% (95% CI, 2.6%–6.5%) in 2007 to 5.5% (95% CI, 4.2%–6.7%) in 2020 (*p* for trend = 0.004).

## 4. Discussion

Using NHANES 2007–2020 data, we found that the age‐standardized prevalence of diabetes among US adults increased from 12.4% to 14.5%, and the age‐standardized prevalence of kidney stones increased from 9.3% to 10.1%. Among adults with diabetes, kidney stone prevalence increased from 14.3% to 16.1% over the same period. The estimated annual number of individuals with both diabetes and kidney stones increased from 269,045 in 2007 to 379,881 in 2020. Exploratory subgroup analyses by sex and race/ethnicity suggested heterogeneity in the trend of kidney stone prevalence among adults with diabetes. This is the first study using NHANES data to systematically evaluate the trends in the prevalence of kidney stones among US adults with and without diabetes. Our findings further support an association between diabetes and higher kidney stone prevalence. These findings may help inform targeted prevention strategies and efforts to reduce disparities in kidney stone burden across subgroups.

In multivariable logistic regression, diabetes was associated with higher odds of kidney stone history after covariate adjustment. Age‐standardized kidney stone prevalence was consistently higher among adults with diabetes than among those without diabetes throughout the study period. These findings are consistent with prior reports [9, 13]. Several biological mechanisms have been proposed. Diabetes‐related hyperglycemia and insulin resistance may shift urinary chemistry (including calcium, oxalate, uric acid, and urine pH), creating a more lithogenic milieu [14–16]. Hyperglycemia may increase urinary uric acid excretion and lower urine pH, thereby favoring uric acid stone formation [15, 17, 18]. Insulin resistance may reduce urinary citrate and impair renal ammonium excretion, further acidifying urine and promoting stone formation [19, 20]. The increasing number of individuals with both conditions underscores the importance of kidney stone prevention and clinical awareness in adults with diabetes.

Sex‐specific subgroup analyses suggested divergent time trends in kidney stone prevalence among adults with diabetes. From 2007 to 2020, kidney stone prevalence increased from 16.6% to 20.2% among men with diabetes and from 11.8% to 12.8% among women with diabetes. Notably, among women with diabetes, prevalence peaked in the 2011–2012 cycle (19.5%) and then declined through 2017–2020, whereas prevalence among men with diabetes continued to rise after 2011. These cycle‐ and subgroup‐specific estimates should be interpreted cautiously given potential sampling variability across NHANES cycles. The drivers of this divergence are unclear and may reflect differences in clinical management, health behaviors, and broader social or policy changes over time. The decline among women with diabetes occurred during a period that temporally coincided with major US policy and nutrition initiatives, including the Affordable Care Act (ACA, 2010) and the MyPlate plan (2011); however, causality cannot be inferred from these data. The ACA, launched in 2010, was implemented with goals that included improving healthcare quality, reducing costs, expanding access to care, and increasing coverage of preventive services, including services relevant to women’s health [21, 22]. Multiple population‐based studies have reported that women use primary and preventive healthcare services more frequently than men [21, 23, 24]. Evidence suggests that ACA‐related coverage expansions and preventive service policies were associated with improved access to preventive care and earlier contact with the health system in some populations, which could contribute to greater opportunities for diabetes prevention, early detection, and comorbidity management, particularly among low‐income women, minority women, and pregnant women [25, 26]. MyPlate (2011) promoted healthier dietary patterns and emphasized adequate water intake. Greater adherence to such guidance has been associated with lower risks of obesity and diabetes [27, 28], and some evidence suggests that women may also adopt nutrition recommendations more readily than men [29]. Together, these policy and nutrition changes provide one possible population‐level context for the observed sex divergence. However, NHANES is cross‐sectional and does not provide longitudinal information on glycemic control, diet/hydration behaviors, or healthcare utilization. As a result, we cannot test these pathways or attribute the observed pattern to any specific initiative, and the interpretation should be viewed as hypothesis‐generating. In parallel with the sustained increase in diabetes prevalence among men, the estimated number of men with both diabetes and kidney stones increased, warranting closer attention. From 2007 to 2020, kidney stone prevalence remained relatively stable among men without diabetes, whereas it increased among women without diabetes. This pattern mirrors the overall increase in kidney stone prevalence among women and suggests that factors beyond diabetes—such as physical inactivity, pregnancy history, hormone therapy, and menopause—may contribute to rising prevalence among women without diabetes [8, 30, 31]. These findings highlight the necessity of effective public health interventions and lifestyle guidance, with particular attention to men with diabetes and women without diabetes, to mitigate the health burden caused by kidney stones.

We observed heterogeneity in kidney stone prevalence trends among US adults with diabetes across race/ethnicity from 2007 to 2020. Among Hispanics with diabetes, kidney stone prevalence increased significantly from 8.7% in 2007–2008 to 14.8% in 2017–2020. In contrast, prevalence among Hispanics without diabetes was relatively stable, suggesting that the increase among Hispanics was concentrated in those with diabetes. Previous studies indicate that US Hispanics have a higher prevalence and incidence of diabetes than the national average [32], and that this excess burden is partly attributed to structural socioeconomic factors, including lower income, higher poverty and uninsurance rates, and reduced access to education and health care resources that support prevention and diabetes self‐management [32, 33]. These social determinants may also influence both kidney stone risk and the likelihood of diagnosis or detection among Hispanic adults with diabetes. NHANES‐based studies have reported independent associations of socioeconomic position and insurance type with nephrolithiasis history: higher stone prevalence among individuals with state‐assisted insurance and lower odds among those with private insurance, even after adjustment [1, 32, 34]. In addition, an increasing prevalence of gout has been reported among Hispanic adults [35]. Diabetes‐related hyperuricemia and gout may represent additional contributors to kidney stone burden in this population. Among non‐Hispanic Whites, kidney stone prevalence increased slightly over time in both the diabetes and nondiabetes groups. Although non‐Hispanic Whites had the lowest diabetes prevalence, they showed the highest kidney stone prevalence among adults with diabetes, ranging from 17.4% to 23.8% over time. This pattern suggests a greater kidney stone burden in this group and may relate to differences in genetic susceptibility or dietary factors [36, 37]. A prior study reported higher urinary calcium excretion and lower urine pH in non‐Hispanic White women than in Black women, corresponding to higher supersaturation for calcium‐containing stones even after adjustment for age, BMI, diet, and diabetes status [38]. Large genetic studies, conducted predominantly in European‐ancestry cohorts, have reported substantial heritability of kidney stone disease and identified susceptibility loci involved in calcium/phosphate handling and calcium‐sensing receptor signaling. Some loci appear to have stronger effects in European‐ than in East Asian–ancestry populations [39]. Given the significant increase in diabetes prevalence among non‐Hispanic Whites, the expanding number of individuals with both diabetes and kidney stones warrant targeted prevention efforts. Among non‐Hispanic Blacks, kidney stone prevalence was relatively stable among adults with diabetes, whereas increases were primarily observed among those without diabetes. This pattern suggests that factors beyond diabetes—such as obesity, high‐salt/high‐sugar diets, lower water intake, and differences in urinary concentration (e.g., creatinine and osmolality)—may be increasingly relevant in this group [40–43]. Overall, these patterns indicate racial/ethnic heterogeneity in the relationship between diabetes status and kidney stone prevalence. Prevention strategies should be tailored to the specific risk profiles of different groups, with particular attention to subgroups with higher observed prevalence, such as Hispanics with diabetes and non‐Hispanic Whites with diabetes.

Using nationally representative NHANES data across multiple cycles, we were able to produce generalizable estimates and describe temporal patterns in kidney stone burden among US adults with diabetes. Nonetheless, several limitations should be noted. First, kidney stone history was self‐reported. Diabetes status was derived from interview and laboratory measures, and reliable classification of diabetes subtype (type 1 vs. type 2) was not available. Therefore, outcome and exposure misclassification is possible. Second, the cross‐sectional design precludes establishing temporality and limits causal inference. Third, although we performed sensitivity analyses with extended covariate adjustment (dietary intake, kidney function (eGFR), serum uric acid, glucose‐lowering medication use, and socioeconomic indicators), residual confounding may persist because some exposures are measured with error and key information is unavailable (e.g., stone composition and diabetes duration/severity). In particular, 24 h dietary recalls primarily reflect short‐term intake and may not capture long‐term dietary patterns, and medication variables in NHANES lack detailed information on dose, duration, and adherence. We also did not assess concentration–response relationships between glycemia markers (HbA1c or fasting plasma glucose) and kidney stone prevalence. In a cross‐sectional setting, these biomarkers are influenced by treatment, and fasting plasma glucose is available only in a fasting subsample with substantial missingness among adults with diabetes, which limits interpretability and statistical power. Fourth, we restricted the analytic sample to adults aged ≥20 years and used complete‐case analyses. Selection bias may occur if missingness is not random. Finally, we conducted multiple subgroup and trend analyses without formal correction for multiple testing. Subgroup‐specific findings should therefore be interpreted cautiously and in the context of the overall pattern of results. This approach is consistent with methodological guidance emphasizing transparent reporting and cautious interpretation in exploratory analyses [44]. Future prospective studies with longitudinal follow‐up, repeated glycemia measurements, and clinically validated outcomes—together with genetic causal‐inference approaches—are warranted to clarify directionality, potential dose–response relationships, and mechanisms linking diabetes and kidney stones. Such data would also help refine prevention and management approaches for groups with higher observed burden.

## 5. Conclusion

From 2007 to 2020, the prevalence of diabetes and kidney stones among US adults increased. Although kidney stone prevalence among adults with diabetes was relatively stable, the rising prevalence of diabetes was accompanied by an increase in the estimated number of adults with both conditions. Subgroup analyses revealed that Hispanics with diabetes experienced a continuous rise in prevalence of kidney stones, whereas men and non‐Hispanic Whites with diabetes continued to have a persistently high prevalence of kidney stones. These findings underscore the urgent need to develop multitiered, individualized prevention strategies for these groups. Future research should explore longitudinal associations and investigate the effectiveness of targeted interventions for this public health challenge.

## Author Contributions

Conceptualization: Wenshuang Li, Xiangfu Zhou, and Bolong Liu. Methodology: Ziqiao Wang, Junlong Huang, and Ruixiang Luo. Investigation: Ziqiao Wang, Junlong Huang, Ruixiang Luo, Fanyu Wu, Ziliang Deng, and Zheng Liu. Data curation: Ziqiao Wang, Junlong Huang, Fanyu Wu, and Ziliang Deng. Formal analysis: Ziqiao Wang, Junlong Huang, Ruixiang Luo, and Zheng Liu. Validation: Ruixiang Luo and Zheng Liu. Supervision: Wenshuang Li, Xiangfu Zhou, and Bolong Liu. Writing – original draft: Ziqiao Wang and Junlong Huang. Writing – review and editing: Wenshuang Li, Xiangfu Zhou, Bolong Liu, and all authors. Ziqiao Wang and Junlong Huang contributed equally to this work and share first authorship.

## Funding

This work was supported by the National Natural Science Foundation of China (Grant 81800666 to Bolong Liu), the Natural Science Foundation of Guangdong Province (Grants 2023A1515010422 and 2025A1515012781 to Bolong Liu, and 2024A1515010461 to Xiangfu Zhou), the Guangzhou Science and Technology Project (Grant 202103000035 to Xiangfu Zhou), the Guangzhou Clinical High‐Tech, Major and Special Technology Projects (Grant 2023P‐TS34 to Xiangfu Zhou), and the Clinical Research Special Fund Sailing Plan Project of the Third Affiliated Hospital of Sun Yat‐sen University (Grant YHJH202205 to Bolong Liu).

## Ethics Statement

This study was approved by the Research Ethics Review Board of the National Center for Health Statistics.

## Consent

Written informed consent was obtained from all participants in the original NHANES survey. No additional informed consent was required for the present study because this was a secondary analysis of publicly available, de‐identified data.

## Conflicts of Interest

The authors declare no conflicts of interest.

## Supporting Information

Additional supporting information can be found online in the Supporting Information section.

## Supporting information


**Supporting Information 1** This file includes the following items: Table S1: Survey‐weighted multivariable logistic regression of the association between diabetes and self‐reported history of kidney stones among US adults in NHANES from 2007 to 2020. Table S2: Survey‐weighted multivariable logistic regression for history of kidney stones with age and BMI modeled as continuous variables in NHANES from 2007 to 2020. Table S3: Sequentially adjusted survey‐weighted logistic regression models for the association between diabetes and history of kidney stones with expanded covariate adjustment in NHANES from 2007 to 2020. Table S4: Trends in age‐standardized prevalence of diabetes in US adults in NHANES from 2007 to 2020. Table S5: Trends in age‐standardized prevalence of kidney stones in US adults by diabetes status in NHANES from 2007 to 2020. Figure S1: Flowchart of the participant enrollment in NHANES from 2007 to 2020. Figure S2: Directed acyclic graph for covariate selection in the analysis of the association between diabetes and history of kidney stones. Figure S3: Trends in age‐standardized prevalence of kidney stones among US adults by sex and race/ethnicity from 2007 to 2020. Figure S4: Trends in age‐standardized prevalence of kidney stones among US adults with or without diabetes by sex from 2007 to 2020. Figure S5: Trends in age‐standardized prevalence of kidney stones among US adults with or without diabetes by race/ethnicity from 2007 to 2020.

**Supporting Information 2** STROBE Checklist: This checklist documents adherence to STROBE reporting guidelines for observational studies.

## Data Availability

The datasets generated during and/or analyzed during the current study are publicly available. This data can be found here: the National Health and Nutrition Examination Survey dataset at https://wwwn.cdc.gov/nchs/nhanes/Default.aspx.
