# Supplementary material for: Trends in Kidney Stone Prevalence Among United States Adults With Diabetes: A Cross‐Sectional Study From the NHANES Database, 2007–2020
Source: J Diabetes Res. 2026 Apr 22;2026:4305574. doi: 10.1155/jdr/4305574 (PMC13100647; doi:10.1155/jdr/4305574)
Supplement: Supplementary file 1 — Supporting Information 1 This file includes the following items: Table S1: Survey‐weighted multivariable logistic regression of the association between diabetes and self‐reported history of kidney stones among US adults in NHANES from 2007 to 2020. Table S2: Survey‐weighted multivariable logistic regression for history of kidney stones with age and BMI modeled as continuous variables in NHANES from 2007 to 2020. Table S3: Sequentially adjusted survey‐weighted logistic regression models for the association between diabetes and history of kidney stones with expanded covariate adjustment in NHANES from 2007 to 2020. Table S4: Trends in age‐standardized prevalence of diabetes in US adults in NHANES from 2007 to 2020. Table S5: Trends in age‐standardized prevalence of kidney stones in US adults by diabetes status in NHANES from 2007 to 2020. Figure S1: Flowchart of the participant enrollment in NHANES from 2007 to 2020. Figure S2: Directed acyclic graph for covariate selection in the analysis of the association between diabetes and history of kidney stones. Figure S3: Trends in age‐standardized prevalence of kidney stones among US adults by sex and race/ethnicity from 2007 to 2020. Figure S4: Trends in age‐standardized prevalence of kidney stones among US adults with or without diabetes by sex from 2007 to 2020. Figure S5: Trends in age‐standardized prevalence of kidney stones among US adults with or without diabetes by race/ethnicity from 2007 to 2020. [file JDR-2026-4305574-s001.docx]

**Supplementary files**

**Supplementary Table 1.** Survey-weighted multivariable logistic regression of the association between diabetes and self-reported history of kidney stones among U.S. adults in NHANES from 2007 to 2020.

**Supplementary Table 2.** Survey-weighted multivariable logistic regression for history of kidney stones with age and BMI modeled as continuous variables in NHANES from 2007 to 2020.

**Supplementary Table 3.** Sequentially adjusted survey-weighted logistic regression models for the association between diabetes and history of kidney stones with expanded covariate adjustment in NHANES from 2007 to 2020.

**Supplementary Table 4.** Trends in age-standardized prevalence of diabetes in U.S. adults in NHANES from 2007 to 2020.

**Supplementary Table 5.** Trends in age-standardized prevalence of kidney stones in U.S. adults by diabetes status in NHANES from 2007 to 2020.

**Supplementary Fig.1** Flowchart of the participant enrollment in NHANES from 2007 to 2020.

**Supplementary Fig.2** Directed acyclic graph for covariate selection in the analysis of the association between diabetes and history of kidney stones.

**Supplementary Fig.3** Trends in age-standardized prevalence of kidney stones among U.S. adults by sex and race/ethnicity from 2007 to 2020.

**Supplementary Fig.4** Trends in age-standardized prevalence of kidney stones among U.S. adults with or without diabetes by sex from 2007 to 2020.

**Supplementary Fig.5** Trends in age-standardized prevalence of kidney stones among U.S. adults with or without diabetes by race/ethnicity from 2007 to 2020.

**Supplementary Table 1.** Survey-weighted multivariable logistic regression of the association between diabetes and self-reported history of kidney stones among U.S. adults in NHANES from 2007 to 2020.

|  | **Odds Ratios (95% Confidence Interval)** | ***P*** |
| --- | --- | --- |
| **Diabetes** |  |  |
| No | ref |  |
| Yes | 1.63 (1.41–1.88) | < 0.001 |
| **Sex** |  |  |
| Male | ref | – |
| Female | 0.73 (0.64–0.83) | < 0.001 |
| **Age, years** |  |  |
| < 40 | ref | – |
| 40–59 | 1.72 (1.50–1.98) | < 0.001 |
| ≥ 60 | 2.03 (1.77–2.33) | < 0.001 |
| **Race** |  |  |
| Non–Hispanic White | ref | – |
| Hispanic | 0.78 (0.69–0.89) | < 0.001 |
| Non–Hispanic Black | 0.43 (0.37–0.50) | < 0.001 |
| Other Race | 0.74 (0.61–0.90) | 0.003 |
| **BMI, kg/m^2^** |  |  |
| < 25 | ref | – |
| 25–30 | 1.14 (0.98–1.33) | 0.089 |
| > 30 | 1.35 (1.12–1.62) | 0.002 |
| **Abdominal obesity** |  |  |
| No | ref | – |
| Yes | 1.29 (1.09–1.52) | 0.004 |
| **Alcohol** |  |  |
| No | ref | – |
| Yes | 0.91 (0.72–1.06) | 0.215 |
| **Smoke** |  |  |
| No | ref | – |
| Yes | 1.12 (0.98–1.29) | 0.104 |

**Supplementary Table 2.** Survey-weighted multivariable logistic regression for history of kidney stones with age and BMI modeled as continuous variables in NHANES from 2007 to 2020.

|  | **Odds Ratios (95% Confidence Interval)** | ***P*** |
| --- | --- | --- |
| **Diabetes** |  |  |
| No | Ref | – |
| Yes | **1.58 (1.36–1.83)** | **<0.001** |
| **Sex** |  |  |
| Male | Ref | – |
| Female | 0.71 (0.63–0.81) | <0.001 |
| **Age (years)** |  |  |
| Per 1–year increase | 1.02 (1.01–1.02) | <0.001 |
| **Race/Ethnicity** |  |  |
| Non–Hispanic White | Ref | – |
| Hispanic | 0.80 (0.71–0.91) | 0.001 |
| Non–Hispanic Black | 0.44 (0.38–0.51) | <0.001 |
| Other Race | 0.76 (0.62–0.92) | 0.006 |
| **BMI (kg/m²)** |  |  |
| Per 1 kg/m² increase | 1.02 (1.01–1.03) | <0.001 |
| **Abdominal obesity** |  |  |
| No | Ref | – |
| Yes | 1.29 (1.12–1.49) | <0.001 |
| **Alcohol use** |  |  |
| No | Ref | – |
| Yes | 0.94 (0.81–1.08) | 0.366 |
| **Smoking status** |  |  |
| No | Ref | – |
| Yes | 1.12 (0.97–1.28) | 0.112 |

| **Supplementary Table 3.** Sequentially adjusted survey-weighted logistic regression models for the association between diabetes and history of kidney stones with expanded covariate adjustment in NHANES from 2007 to 2020.   \|  \|  \| Diabetes \| \| \| --- \| --- \| --- \| --- \| \|  \|  \| No \| Yes \| \| Model 1 \| OR (95% CI) \| Ref \| 2.22 (1.95–2.53) \| \| *P* \|  \| <0.001 \| \| Model 2 \| OR (95% CI) \| Ref \| 1.63 (1.41–1.88) \| \| *P* \|  \| <0.001 \| \| Model 3 \| OR (95% CI) \| Ref \| 1.50 (1.13–1.99) \| \| *P* \|  \| 0.006 \|   Abbreviations: OR, odds ratio; CI, confidence interval; eGFR, estimated glomerular filtration rate; PIR, poverty income ratio.  Model specification:  Model 1: Crude (unadjusted) model.  Model 2 (primary confounder–adjusted model): Adjusted for age, sex (male/female), race/ethnicity, BMI, abdominal obesity, alcohol use, and smoking status.  Model 3 (expanded adjustment; sensitivity analysis): Additionally adjusted for dietary intake (total energy, water, sodium, calcium, protein, and sugar), kidney function (eGFR), serum uric acid, glucose–lowering medication use, and PIR. |
| --- | --- | --- | --- | --- | --- | --- | --- | --- | --- | --- | --- | --- | --- | --- | --- | --- | --- | --- | --- | --- | --- | --- | --- | --- | --- | --- | --- | --- | --- |

| **Supplementary Table 4.** Trends in age-standardized prevalence of diabetes in U.S. adults in NHANES from 2007 to 2020. | | | | | | | |
| --- | --- | --- | --- | --- | --- | --- | --- |
| Characteristic | Prevalence, % (95% Confidence Interval) | | | | | | *P* for trend |
|  | 2007–2008 | 2009–2010 | 2011–2012 | 2013–2014 | 2015–2016 | 2017–2020 |  |
| Overall | 12.4 (11.1–13.9) | 12.5 (11.3–13.8) | 12.4 (11.1–13.8) | 13.1 (12.1–14.1) | 14.4 (12.8–16.0) | 14.5 (13.4–15.6) | 0.004 |
| Male | 13.3 (11.8–14.8) | 13.5 (11.7–15.3) | 12.5 (10.5–14.6) | 13.9 (12.1–15.6) | 16.1 (13.7–18.4) | 16.0 (14.4–17.6) | 0.003 |
| Female | 11.8 (10.0–13.6) | 11.5 (10.2–12.9) | 12.3 (10.5–14.1) | 12.4 (11.0–13.8) | 12.8 (11.2–14.3) | 13.1 (11.7–14.5) | 0.154 |
| Hispanic | 16.6 (14.4–18.8) | 20.2 (17.1–23.3) | 19.0 (16.0–21.9) | 18.5 (16.2–20.8) | 22.0 (19.4–24.7) | 19.2 (17.2–21.3) | 0.205 |
| Non–Hispanic White | 10.8 (9.0–12.7) | 10.4 (8.9–11.8) | 9.6 (8.2–11.0) | 11.3 (10.1–12.6) | 12.0 (10.2–13.9) | 12.6 (11.1–14.0) | 0.035 |
| Non–Hispanic Black | 22.4 (20.0–24.8) | 18.3 (15.7–20.8) | 21.3 (18.2–24.4) | 18.7 (17.0–20.3) | 20.7 (18.4–23.0) | 18.7 (16.9–20.5) | 0.126 |

*P* < 0.05 was considered significant.

| **Supplementary Table 5.** Trends in age-standardized prevalence of kidney stones in U.S. adults by diabetes status in NHANES from 2007 to 2020. | | | | | | | |
| --- | --- | --- | --- | --- | --- | --- | --- |
| Characteristic | Prevalence, % (95% Confidence Interval) | | | | | | *P* for trend |
|  | 2007–2008 | 2009–2010 | 2011–2012 | 2013–2014 | 2015–2016 | 2017–2020 |  |
| **Overall** | 9.3 (8.3–10.3) | 9.4 (8.3–10.5) | 8.4 (7.2–9.7) | 10.5 (9.4–11.6) | 12.0 (10.6–13.3) | 10.1 (9.2–11.1) | 0.008 |
| With Diabetes | 14.3 (10.9–17.7) | 13.8 (10.1–17.5) | 15.2 (10.9–19.5) | 16.0 (13.0–18.9) | 18.4 (14.3–22.5) | 16.1 (10.3–22.0) | 0.446 |
| Without Diabetes | 8.5 (7.4–9.7) | 8.8 (7.5–10.1) | 7.7 (6.3–9.0) | 9.3 (8.0–10.5) | 10.8 (9.6–12.0) | 9.4 (8.2–10.6) | 0.028 |
| **Male** | 12.3 (10.8–13.8) | 10.9 (9.2–12.6) | 8.7 (7.1–10.3) | 11.7 (9.9–13.4) | 13.9 (11.8–16.0) | 11.1 (9.5–12.8) | 0.489 |
| With Diabetes | 16.6 (12.2–20.9) | 13.4 (7.2–19.6) | 10.5 (5.6–15.5) | 19.0 (13.4–24.5) | 20.9 (15.7–26.0) | 20.2 (8.9–31.5) | 0.527 |
| Without Diabetes | 11.4 (9.7–13.2) | 10.6 (8.6–12.6) | 8.1 (6.4–9.8) | 10.2 (8.3–12.2) | 12.6 (10.7–14.5) | 10.4 (8.6–12.2) | 0.748 |
| **Female** | 6.7 (5.4–8.0) | 7.9 (6.7–9.2) | 8.2 (6.4–10.0) | 9.4 (8.1–10.8) | 10.2 (8.4–12.0) | 9.2 (7.8–10.6) | 0.003 |
| With Diabetes | 11.8 (6.5–17.1) | 14.4 (9.4–19.4) | 19.5 (11.8–27.2) | 13.1 (8.2–18.0) | 15.4 (10.0–20.8) | 12.8 (8.2–17.4) | 0.848 |
| Without Diabetes | 6.0 (4.6–7.4) | 7.2 (5.7–8.6) | 7.3 (5.4–9.1) | 8.6 (7.2–10.0) | 9.3 (7.3–11.4) | 8.6 (7.1–10.1) | 0.001 |
| **Hispanic** | 8.4 (6.9–9.8) | 8.3 (7.1–9.6) | 10.9 (9.1–12.7) | 8.7 (6.9–10.5) | 10.9 (9.0–12.7) | 10.1 (8.5–11.7) | 0.036 |
| With Diabetes | 8.7 (5.3–12.0) | 9.7 (7.0–12.3) | 14.0 (6.5–21.5) | 13.5 (9.0–17.9) | 15.3 (12.5–18.2) | 14.8 (10.3–19.3) | 0.017 |
| Without Diabetes | 7.9 (6.2–9.6) | 8.0 (6.4–9.5) | 10.7 (8.8–12.6) | 6.4 (4.9–7.8) | 9.2 (7.1–11.3) | 9.1 (7.3–11.0) | 0.202 |
| **Non–Hispanic White** | 10.3 (9.0–11.6) | 10.4 (8.8–11.9) | 8.7 (7.0–10.3) | 12.1 (10.6–13.7) | 13.5 (11.7–15.3) | 10.9 (9.5–12.3) | 0.038 |
| With Diabetes | 17.4 (12.6–22.2) | 17.7 (10.2–25.2) | 19.5 (13.1–25.8) | 17.5 (12.4–22.6) | 23.8 (17.6–30.0) | 21.7 (10.1–33.4) | 0.468 |
| Without Diabetes | 9.3 (7.9–10.7) | 9.8 (8.1–11.6) | 8.0 (6.3–9.6) | 11.0 (9.3–12.6) | 12.1 (10.3–13.9) | 10.0 (8.4–11.6) | 0.093 |
| **Non–Hispanic Black** | 4.8 (3.4–6.3) | 5.4 (3.9–7.0) | 4.8 (3.6–6.1) | 5.7 (4.1–7.4) | 6.2 (4.7–7.8) | 6.1 (4.9–7.3) | 0.039 |
| With Diabetes | 7.7 (3.5–11.9) | 6.9 (3.4–10.3) | 10.8 (4.3–17.2) | 9.8 (2.3–17.2) | 7.6 (3.7–11.5) | 6.9 (3.8–9.9) | 0.775 |
| Without Diabetes | 4.5 (2.6–6.5) | 4.6 (3.1–6.0) | 3.3 (2.3–4.3) | 5.5 (3.8–7.1) | 5.8 (3.8–7.8) | 5.5 (4.2–6.7) | 0.004 |

*P* < 0.05 was considered significant.

**Supplementary Fig.1** Flowchart of the participant enrollment in NHANES from 2007 to 2020.


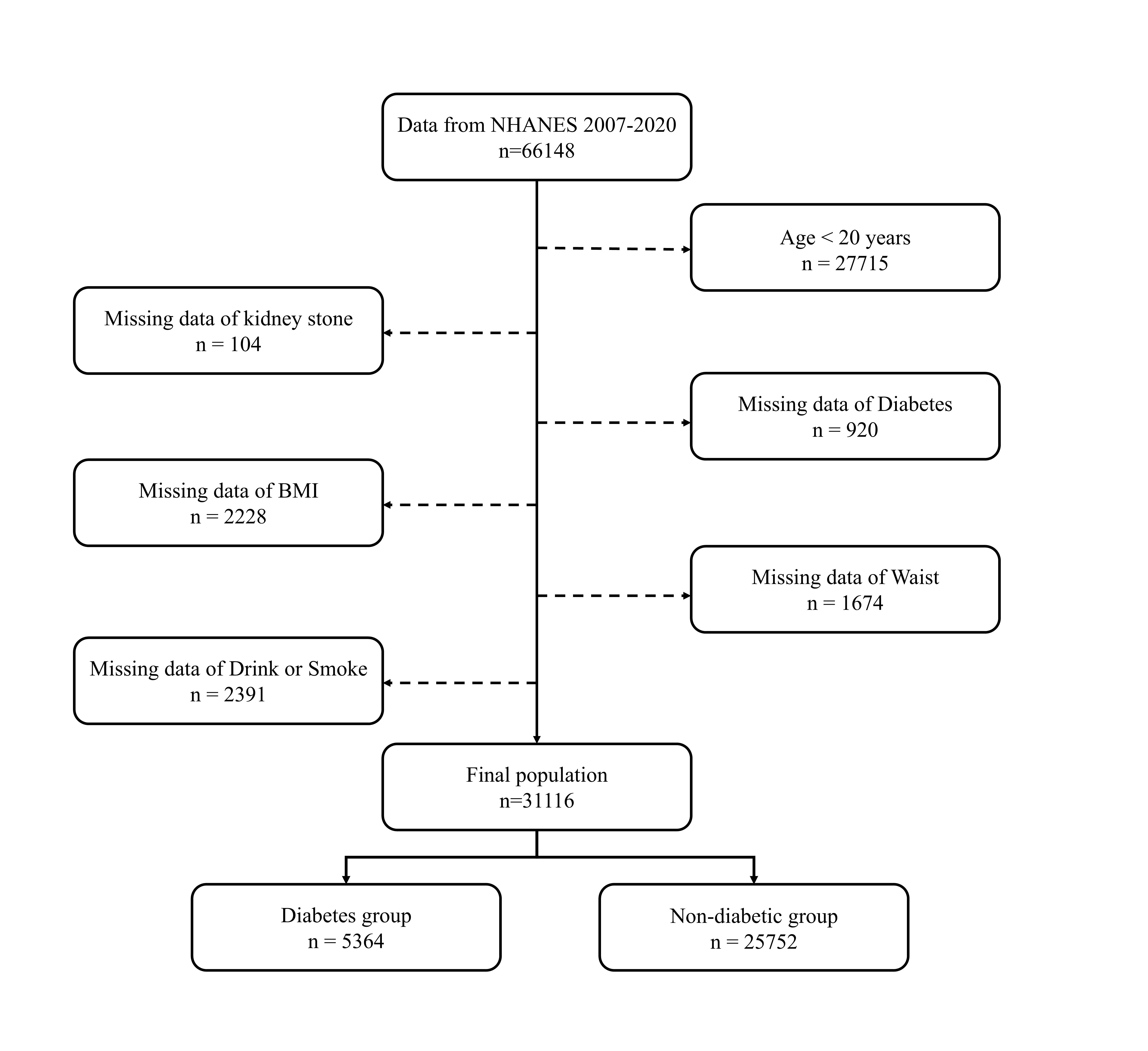


Abbreviations: NHANES, National Health and Nutrition Examination Survey; BMI, Body mass index

**Supplementary Fig.2** Directed acyclic graph for covariate selection in the analysis of the association between diabetes and history of kidney stones.

**
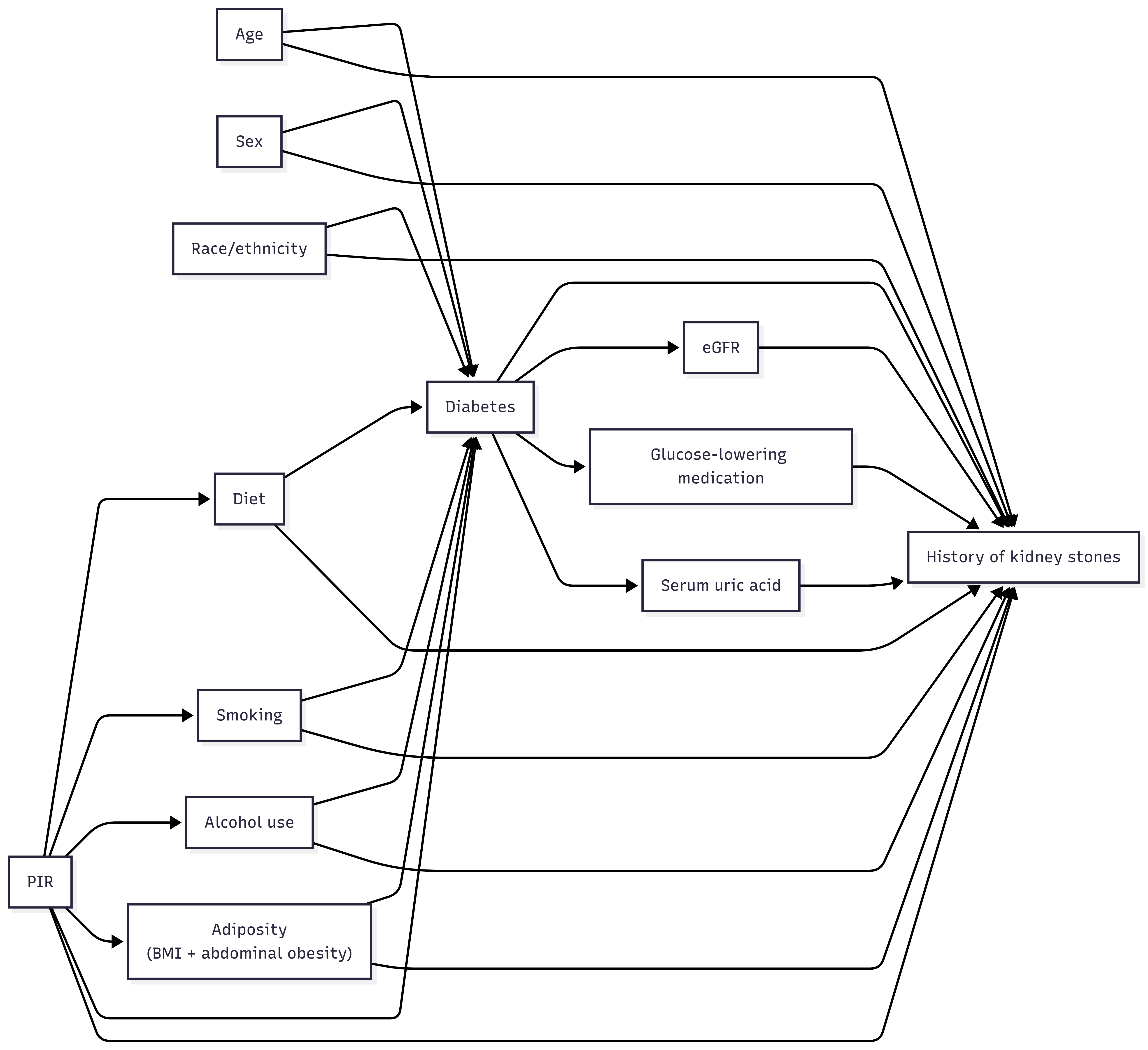
**

DAG, directed acyclic graph; BMI, body mass index; PIR, poverty income ratio; eGFR, estimated glomerular filtration rate.

**Supplementary** **Fig.3** Trends in age-standardized prevalence of kidney stones among U.S. adults by sex and race/ethnicity from 2007 to 2020.


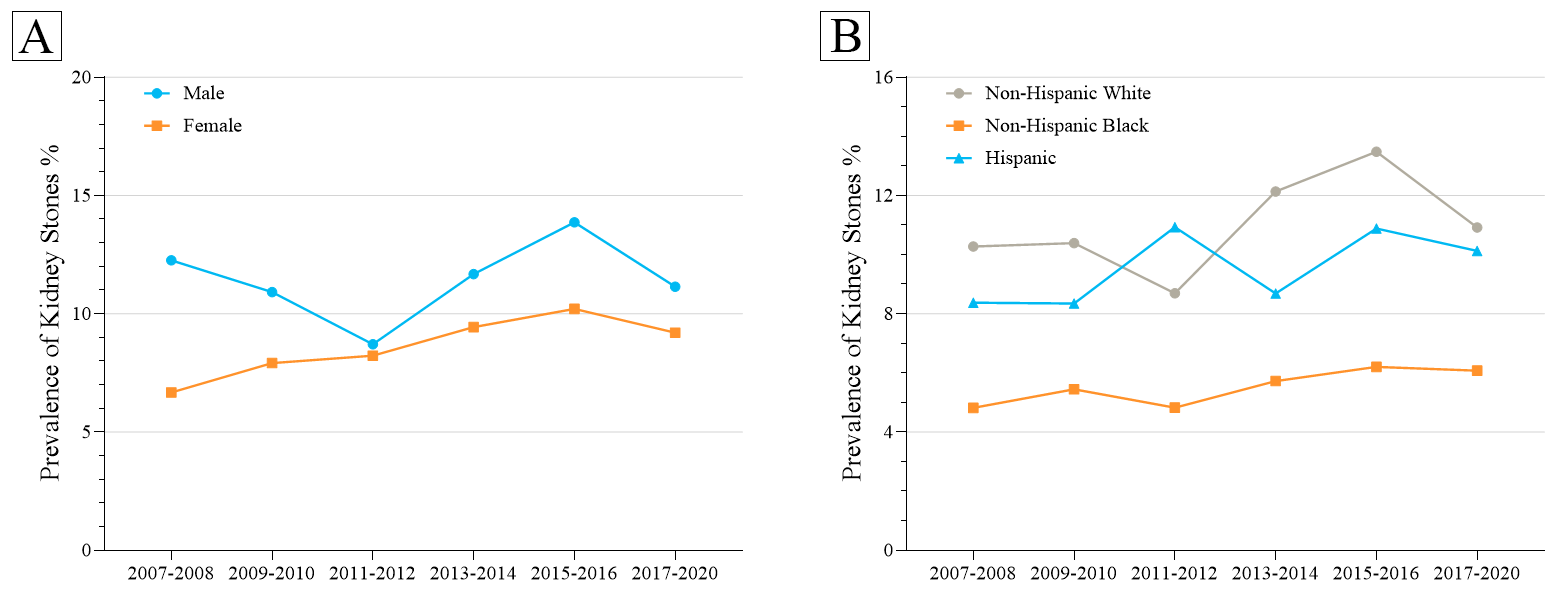


A: Trends in age-standardized prevalence of kidney stones among U.S. adults by sex from 2007 to 2020; B: Trends in age-standardized prevalence of kidney stones among U.S. adults by race/ethnicity from 2007 to 2020.

**Supplementary Fig.4** Trends in age-standardized prevalence of kidney stones among U.S. adults with or without diabetes by sex from 2007 to 2020.


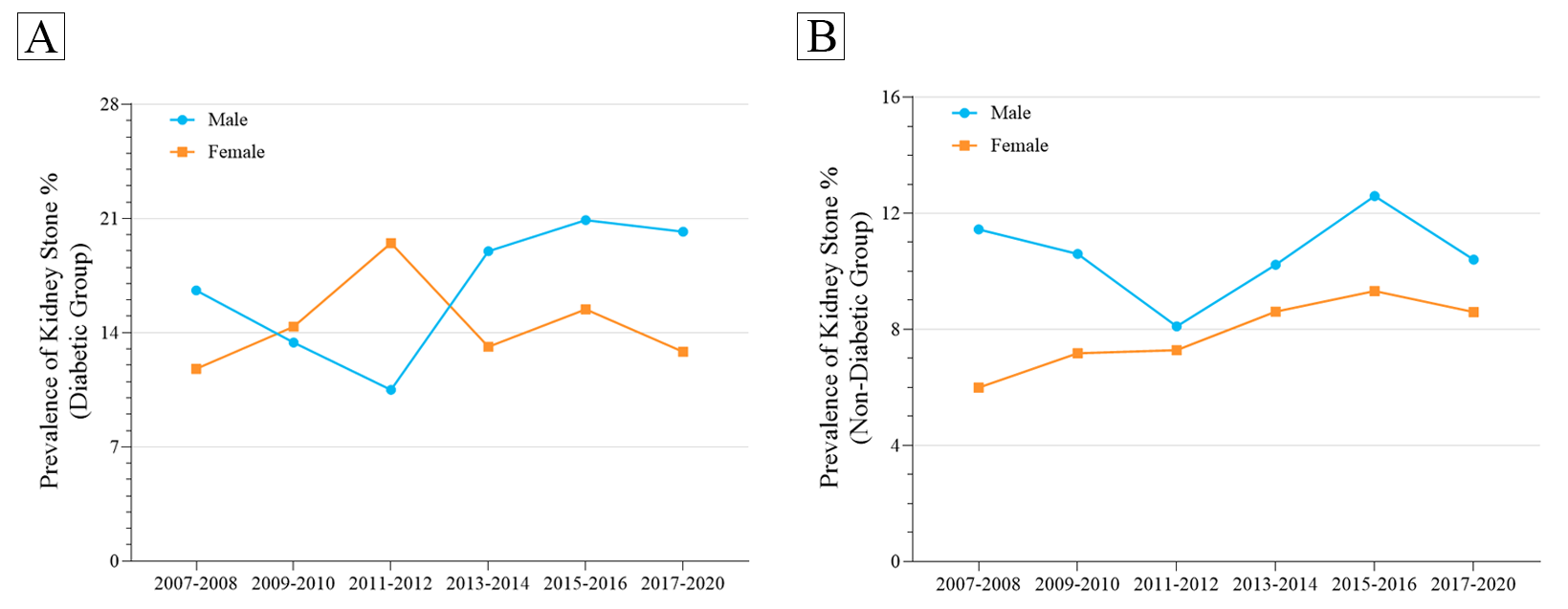


A: Trends in age-standardized prevalence of kidney stones among U.S. adults with diabetes by sex from 2007 to 2020; B: Trends in age-standardized prevalence of kidney stones among U.S. adults without diabetes by sex from 2007 to 2020.

**Supplementary Fig.5** Trends in age-standardized prevalence of kidney stones among U.S. adults with or without diabetes by race/ethnicity from 2007 to 2020.


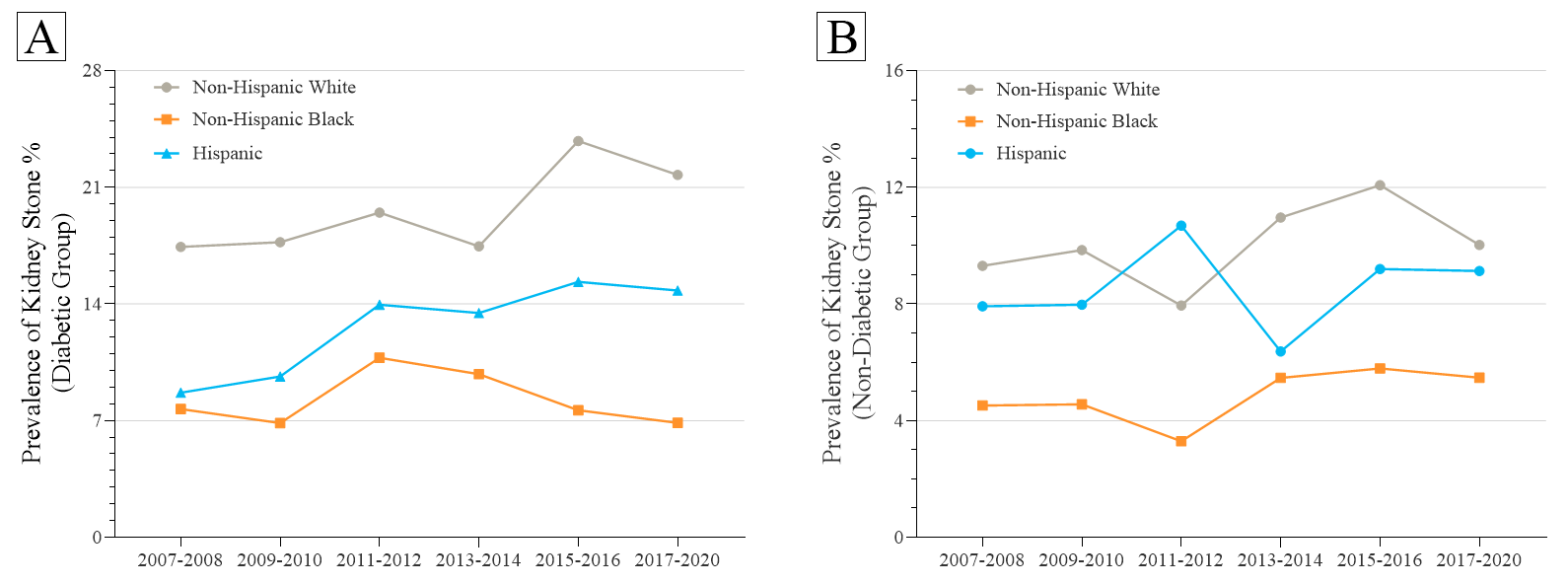


A: Trends in age-standardized prevalence of kidney stones among U.S. adults with diabetes by race/ethnicity from 2007 to 2020; B: Trends in age-standardized prevalence of kidney stones among U.S. adults without diabetes by race/ethnicity from 2007 to 2020.
