# Supplementary material for: Trends in Kidney Stone Prevalence Among United States Adults With Diabetes: A Cross‐Sectional Study From the NHANES Database, 2007–2020
Source: J Diabetes Res. 2026 Apr 22;2026:4305574. doi: 10.1155/jdr/4305574 (PMC13100647; doi:10.1155/jdr/4305574)
Supplement: Supplementary file 2 — Supporting Information 2 STROBE Checklist: This checklist documents adherence to STROBE reporting guidelines for observational studies. [file JDR-2026-4305574-s002.docx]

STROBE Statement—checklist of items that should be included in reports of observational studies

|  | Item No. | Recommendation | Page  No. | Relevant text from manuscript |
| --- | --- | --- | --- | --- |
| **Title and abstract** | 1 | (*a*) Indicate the study’s design with a commonly used term in the title or the abstract | 1 | Trends in Kidney Stone Prevalence among United States Adults with Diabetes: A Cross-Sectional Study from the NHANES Database, 2007–2020 |
|  |  | (*b*) Provide in the abstract an informative and balanced summary of what was done and what was found | 3-4 | From 2007 to 2020, the prevalence of diabetes significantly increased. Diabetic Hispanics experienced a continuous rise in kidney stone prevalence, while diabetic men and non-Hispanic Whites maintained persistently high prevalence. These subgroups may warrant targeted prevention and clinical attention. |
| Introduction | | | |  |
| Background/rationale | 2 | Explain the scientific background and rationale for the investigation being reported | 4 | Although the association between diabetes and kidney stones is well documented, temporal trends in kidney stone prevalence among adults with diabetes remain incompletely characterized. |
| Objectives | 3 | State specific objectives, including any prespecified hypotheses | 4-5 | Using this nationally representative dataset, we analysed temporal trends in the prevalence of kidney stones among U.S. adults with diabetes from 2007 to 2020, explored factors associated with kidney stones, and evaluated the related public health implications. |
| Methods | | | |  |
| Study design | 4 | Present key elements of study design early in the paper | 5 | We conducted a cross-sectional analysis using data from six NHANES cycles from 2007 to 2020. |
| Setting | 5 | Describe the setting, locations, and relevant dates, including periods of recruitment, exposure, follow-up, and data collection | 5 | We conducted a cross-sectional analysis using data from six NHANES cycles from 2007 to 2020. |
| Participants | 6 | (*a*) *Cohort study*—Give the eligibility criteria, and the sources and methods of selection of participants. Describe methods of follow-up  *Case-control study*—Give the eligibility criteria, and the sources and methods of case ascertainment and control selection. Give the rationale for the choice of cases and controls  *Cross-sectional study*—Give the eligibility criteria, and the sources and methods of selection of participants | 5 | We conducted a cross-sectional analysis using data from six NHANES cycles from 2007 to 2020. |
|  |  | (*b*) *Cohort study*—For matched studies, give matching criteria and number of exposed and unexposed  *Case-control study*—For matched studies, give matching criteria and the number of controls per case |  |  |
| Variables | 7 | Clearly define all outcomes, exposures, predictors, potential confounders, and effect modifiers. Give diagnostic criteria, if applicable | 5 | Diabetes was defined as a self-reported diagnosis, fasting blood glucose (FBG) ≥126 mg/dL, or HbA1c ≥6.5%11. Kidney stones were identified by a positive response to the question “Ever had kidney stones?” Participants who answered “yes” were considered to have a history of kidney stones. |
| Data sources/ measurement | 8* | For each variable of interest, give sources of data and details of methods of assessment (measurement). Describe comparability of assessment methods if there is more than one group | *5* | Diabetes was defined as a self-reported diagnosis, fasting blood glucose (FBG) ≥126 mg/dL, or HbA1c ≥6.5%11. Kidney stones were identified by a positive response to the question “Ever had kidney stones?” Participants who answered “yes” were considered to have a history of kidney stones. |
| Bias | 9 | Describe any efforts to address potential sources of bias | 14 | First, kidney stone history was self-reported. Diabetes status was derived from interview and laboratory measures, and reliable classification of diabetes subtype (type 1 vs. type 2) was not available. Therefore, outcome and exposure misclassification is possible. Second, the cross-sectional design precludes establishing temporality and limits causal inference. Third, although we performed sensitivity analyses with extended covariate adjustment (dietary intake, kidney function (eGFR), serum uric acid, glucose-lowering medication use, and socioeconomic indicators), residual confounding may persist because some exposures are measured with error and key information is unavailable (e.g., stone composition and diabetes duration/severity). In particular, 24-hour dietary recalls primarily reflect short-term intake and may not capture long-term dietary patterns, and medication variables in NHANES lack detailed information on dose, duration, and adherence. We also did not assess concentration–response relationships between glycaemic markers (HbA1c or fasting plasma glucose) and kidney stone prevalence. In a cross-sectional setting, these biomarkers are influenced by treatment, and fasting plasma glucose is available only in a fasting subsample with substantial missingness among adults with diabetes, which limits interpretability and statistical power. Fourth, we restricted the analytic sample to adults aged ≥20 years and used complete-case analyses. Selection bias may occur if missingness is not random. Finally, we conducted multiple subgroup and trend analyses without formal correction for multiple testing. Subgroup-specific findings should therefore be interpreted cautiously and in the context of the overall pattern of results. |
| Study size | 10 | Explain how the study size was arrived at | 5 | The final sample comprised 31,116 participants, including 5,364 individuals with diabetes and 25,752 individuals without diabetes. |

Continued on next page

| Quantitative variables | 11 | Explain how quantitative variables were handled in the analyses. If applicable, describe which groupings were chosen and why | 6 | Age was grouped into three categories: 20–39, 40–59, and ≥60 years. Gender was classified as men or women. Race was categorized into four groups: Hispanic, non-Hispanic White, non-Hispanic Black, and Other. Body mass index (BMI) was categorized as < 25 kg/m², 25–30 kg/m², or ≥30 kg/m². Abdominal obesity was defined as a waist circumference ≥102 cm for men or ≥88 cm for women12. Smoking status was determined by whether participants had smoked at least 100 cigarettes in their life. Alcohol use was defined as having consumed at least 12 drinks of any alcoholic beverage in their lifetime. Dietary intake variables (total energy, total water, sodium, calcium, protein, and total sugars) were obtained from NHANES 24-hour dietary recall data and summarized as the mean of two recall days when available (otherwise using the single available day). Kidney function was assessed by estimated glomerular filtration rate (eGFR), calculated from serum creatinine using the CKD-EPI 2021 equation. Serum uric acid was obtained from laboratory measurements. Socioeconomic status was represented by the poverty income ratio (PIR). Glucose-lowering medication use was defined as current use of insulin and/or oral hypoglycemic agents (yes/no). |
| --- | --- | --- | --- | --- |
| Statistical methods | 12 | (*a*) Describe all statistical methods, including those used to control for confounding | 6 | We used weighted multivariable logistic regression to assess the association between diabetes and kidney stones. We constructed sequential models: Model 1 (unadjusted); Model 2 (primary adjusted model) adjusted for age group, sex, race/ethnicity, BMI category, abdominal obesity, alcohol use, and smoking status; and Model 3 (expanded adjustment; sensitivity analysis) further adjusted for dietary intake variables (total energy, total water, sodium, calcium, protein, and total sugars), eGFR, serum uric acid, PIR, and glucose-lowering medication use (insulin and/or oral hypoglycemic agents; yes/no). Model 3 was conducted as an expanded-adjustment sensitivity analysis in the subset with available data for the additional covariates.  Temporal trends were evaluated by treating NHANES cycles as a continuous variable in survey-weighted regression models, with trend significance assessed by P for trend. |
|  |  | (*b*) Describe any methods used to examine subgroups and interactions | 7 | Subgroup analyses by sex and race/ethnicity were conducted to explore potential heterogeneity and were considered secondary and exploratory. |
|  |  | (*c*) Explain how missing data were addressed | 5 | Participants were excluded based on the following criteria: age under 20 years, missing data for diabetes status or kidney stones, or incomplete covariate information. |
|  |  | (*d*) *Cohort study*—If applicable, explain how loss to follow-up was addressed  *Case-control study*—If applicable, explain how matching of cases and controls was addressed  *Cross-sectional study*—If applicable, describe analytical methods taking account of sampling strategy | 6 | This study used NHANES sampling weights and design variables to estimate the prevalence of diabetes and kidney stones, ensuring nationally representative estimates for the U.S. non-institutionalized population. |
|  |  | (*e*) Describe any sensitivity analyses | 6-7 | We used weighted multivariable logistic regression to assess the association between diabetes and kidney stones. We constructed sequential models: Model 1 (unadjusted); Model 2 (primary adjusted model) adjusted for age group, sex, race/ethnicity, BMI category, abdominal obesity, alcohol use, and smoking status; and Model 3 (expanded adjustment; sensitivity analysis) further adjusted for dietary intake variables (total energy, total water, sodium, calcium, protein, and total sugars), eGFR, serum uric acid, PIR, and glucose-lowering medication use (insulin and/or oral hypoglycemic agents; yes/no). Model 3 was conducted as an expanded-adjustment sensitivity analysis in the subset with available data for the additional covariates. |
| Results | | | | |
| Participants | 13* | (a) Report numbers of individuals at each stage of study—eg numbers potentially eligible, examined for eligibility, confirmed eligible, included in the study, completing follow-up, and analysed | 5 | The final sample comprised 31,116 participants, including 5,364 individuals with diabetes and 25,752 individuals without diabetes |
|  |  | (b) Give reasons for non-participation at each stage | 5 | Participants were excluded based on the following criteria: age under 20 years, missing data for diabetes status or kidney stones, or incomplete covariate information. |
|  |  | (c) Consider use of a flow diagram | 5 | A flowchart detailing participant enrollment is provided in Supplementary Fig.1. |
| Descriptive data | 14* | (a) Give characteristics of study participants (eg demographic, clinical, social) and information on exposures and potential confounders | 6 | 3.1Baseline Characteristics of Participants |
|  |  | (b) Indicate number of participants with missing data for each variable of interest | 5 | A flowchart detailing participant enrollment is provided in Supplementary Fig.1. |
|  |  | (c) *Cohort study*—Summarise follow-up time (eg, average and total amount) |  |  |
| Outcome data | 15* | *Cohort study*—Report numbers of outcome events or summary measures over time |  |  |
|  |  | *Case-control study—*Report numbers in each exposure category, or summary measures of exposure |  |  |
|  |  | *Cross-sectional study—*Report numbers of outcome events or summary measures | *5* | The final sample comprised 31,116 participants, including 5,364 individuals with diabetes and 25,752 individuals without diabetes. |
| Main results | 16 | (*a*) Give unadjusted estimates and, if applicable, confounder-adjusted estimates and their precision (eg, 95% confidence interval). Make clear which confounders were adjusted for and why they were included | 7 | We used weighted multivariable logistic regression to assess the association between diabetes and kidney stones. We constructed sequential models: Model 1 (unadjusted); Model 2 (primary adjusted model) adjusted for age group, sex, race/ethnicity, BMI category, abdominal obesity, alcohol use, and smoking status; and Model 3 (expanded adjustment; sensitivity analysis) further adjusted for dietary intake variables (total energy, total water, sodium, calcium, protein, and total sugars), eGFR, serum uric acid, PIR, and glucose-lowering medication use (insulin and/or oral hypoglycemic agents; yes/no). Model 3 was conducted as an expanded-adjustment sensitivity analysis in the subset with available data for the additional covariates. |
|  |  | (*b*) Report category boundaries when continuous variables were categorized | 6 | Age was grouped into three categories: 20–39, 40–59, and ≥60 years. Sex was classified as men or women. Race was categorized into four groups: Hispanic, non-Hispanic White, non-Hispanic Black, and Other. Body mass index (BMI) was categorized as < 25 kg/m², 25–30 kg/m², or ≥30 kg/m². Abdominal obesity was defined as a waist circumference ≥102 cm for men or ≥88 cm for women12. Smoking status was determined by whether participants had smoked at least 100 cigarettes in their life. Alcohol use was defined as having consumed at least 12 drinks of any alcoholic beverage in their lifetime. |
|  |  | (*c*) If relevant, consider translating estimates of relative risk into absolute risk for a meaningful time period |  |  |

Continued on next page

| Other analyses | 17 | Report other analyses done—eg analyses of subgroups and interactions, and sensitivity analyses | 7-10 |  |
| --- | --- | --- | --- | --- |
| Discussion | | | | |
| Key results | 18 | Summarise key results with reference to study objectives | 12 | Using NHANES 2007–2020 data, we found that the age-standardized prevalence of diabetes among U.S. adults increased from 12.4% to 14.5%, and the age-standardized prevalence of kidney stones increased from 9.3% to 10.1%. Among adults with diabetes, kidney stone prevalence increased from 14.3% to 16.1% over the same period. The estimated annual number of individuals with both diabetes and kidney stones increased from 269,045 in 2007 to 379,881 in 2020. Exploratory subgroup analyses by sex and race/ethnicity suggested heterogeneity in kidney stone prevalence among adults with diabetes. |
| Limitations | 19 | Discuss limitations of the study, taking into account sources of potential bias or imprecision. Discuss both direction and magnitude of any potential bias | 16 | Nonetheless, several limitations should be noted. First, kidney stone history was self-reported. Diabetes status was derived from interview and laboratory measures, and reliable classification of diabetes subtype (type 1 vs. type 2) was not available. Therefore, outcome and exposure misclassification is possible. Second, the cross-sectional design precludes establishing temporality and limits causal inference. Third, although we performed sensitivity analyses with extended covariate adjustment (dietary intake, kidney function (eGFR), serum uric acid, glucose-lowering medication use, and socioeconomic indicators), residual confounding may persist because some exposures are measured with error and key information is unavailable (e.g., stone composition and diabetes duration/severity). In particular, 24-hour dietary recalls primarily reflect short-term intake and may not capture long-term dietary patterns, and medication variables in NHANES lack detailed information on dose, duration, and adherence. We also did not assess concentration–response relationships between glycaemic markers (HbA1c or fasting plasma glucose) and kidney stone prevalence. In a cross-sectional setting, these biomarkers are influenced by treatment, and fasting plasma glucose is available only in a fasting subsample with substantial missingness among adults with diabetes, which limits interpretability and statistical power. Fourth, we restricted the analytic sample to adults aged ≥20 years and used complete-case analyses. Selection bias may occur if missingness is not random. |
| Interpretation | 20 | Give a cautious overall interpretation of results considering objectives, limitations, multiplicity of analyses, results from similar studies, and other relevant evidence |  |  |
| Generalisability | 21 | Discuss the generalisability (external validity) of the study results | 16 | Using nationally representative NHANES data across multiple cycles, we were able to produce generalizable estimates and describe temporal patterns in kidney stone burden among U.S. adults with diabetes. |
| Other information | |  | | |
| Funding | 22 | Give the source of funding and the role of the funders for the present study and, if applicable, for the original study on which the present article is based | 18 | This study was funded by the National Natural Science Foundation of China (grant numbers: 82170786 and 81670688 to Xiangfu Zhou) |

*Give information separately for cases and controls in case-control studies and, if applicable, for exposed and unexposed groups in cohort and cross-sectional studies.

**Note:** An Explanation and Elaboration article discusses each checklist item and gives methodological background and published examples of transparent reporting. The STROBE checklist is best used in conjunction with this article (freely available on the Web sites of PLoS Medicine at http://www.plosmedicine.org/, Annals of Internal Medicine at http://www.annals.org/, and Epidemiology at http://www.epidem.com/). Information on the STROBE Initiative is available at www.strobe-statement.org.
